# The emerging role of extracellular vesicles in fungi: a double-edged sword

**DOI:** 10.3389/fmicb.2023.1216895

**Published:** 2023-07-18

**Authors:** Yi Lai, Bowei Jiang, Fangpeng Hou, Xinhong Huang, Baodian Ling, Hongfei Lu, Tianyu Zhong, Junyun Huang

**Affiliations:** ^1^The First School of Clinical Medicine, Gannan Medical University, Ganzhou, Jiangxi, China; ^2^Laboratory Medicine, First Affiliated Hospital of Gannan Medical University, Ganzhou, Jiangxi, China

**Keywords:** fungus, fungal infection, extracellular vesicles, fungal-host interaction, immunomodulation, drug carrier, vaccine

## Abstract

Fungi are eukaryotic microorganisms found in nature, which can invade the human body and cause tissue damage, inflammatory reactions, organ dysfunctions, and diseases. These diseases can severely damage the patient’s body systems and functions, leading to a range of clinical symptoms that can be life-threatening. As the incidence of invasive fungal infections has progressively increased in the recent years, a wealth of evidence has confirmed the “double-edged sword” role of fungal extracellular vesicles (EVs) in intercellular communication and pathogen-host interactions. Fungal EVs act as mediators of cellular communication, affecting fungal-host cell interactions, delivering virulence factors, and promoting infection. Fungal EVs can also have an induced protective effect, affecting fungal growth and stimulating adaptive immune responses. By integrating recent studies, we discuss the role of EVs in fungi, providing strong theoretical support for the early prevention and treatment of invasive fungal infections. Finally, we highlight the feasibility of using fungal EVs as drug carriers and in vaccine development.

## 1. Introduction

Fungi are a class of eukaryotic microorganisms with a membrane-enclosed nucleus and additional organelles. Fungi exist in nature in a parasitic or saprophytic form and are capable of asexual or sexual reproduction (Sun et al., 2020). Among the millions of documented fungal species, approximately 300 are known to cause human diseases (Casadevall, 2022). In recent years, with the application of large doses of antibiotics, hormones, and immunosuppressants, an increasing number of fungi have invaded the human organism, where they have grown and multiplied in tissues, organs, or in the blood. In addition to their main infectious diseases (also known as invasive fungal diseases, IFD; Lamoth et al., 2022), they can cause inflammatory reactions and tissue damage. National and international epidemiological studies have shown that the overall incidence of IFD has been progressively increasing each year (Rayens et al., 2022). Nevertheless, the outcome of these fungal infections has often been overlooked. For example, the World Health Organization does not have any program on fungal infections and most public health facilities rarely conduct fungal surveillance procedures. In addition, most physicians handling fungal infections have no formal training in medical mycology. Moreover, severe fungal infections remain the leading cause of HIV or AIDS-related deaths worldwide (Limper et al., 2017; Tan et al., 2020).

Extracellular vesicles (EVs) are secreted by the vast majority of the cells, having an irreducible phospholipid bilayer structure. Due to the lack of reliable specific markers of their subcellular origin, the International Society for Extracellular Vesicles (ISEV) recommends their description in experimental systems to be based on specific physicochemical properties of EVs, such as their size, biochemical composition, and conditions describing their origin (Théry et al., 2018). Based on their size, they are classified as small EVs (sEVs; Liu et al., 2022), medium EVs, and/or large EVs (Gabrielli et al., 2022). EVs that are classified according to their biogenesis as originating from cells undergoing apoptosis are called apoptotic vesicles (with a diameter of 50–1,000 nm). EVs released from living cells include microvesicles (with a diameter of 50–1,000 nm) and exosomes (30–150 nm). Because of their overlapping sizes, surface markers, and the lack of proteins restricted to specific populations, the generic term “extracellular vesicles (EVs)” will be used in this review, in accordance with the latest ISEV guidelines.

## 2. Biogenesis of fungal EVs

**Figure 1:**
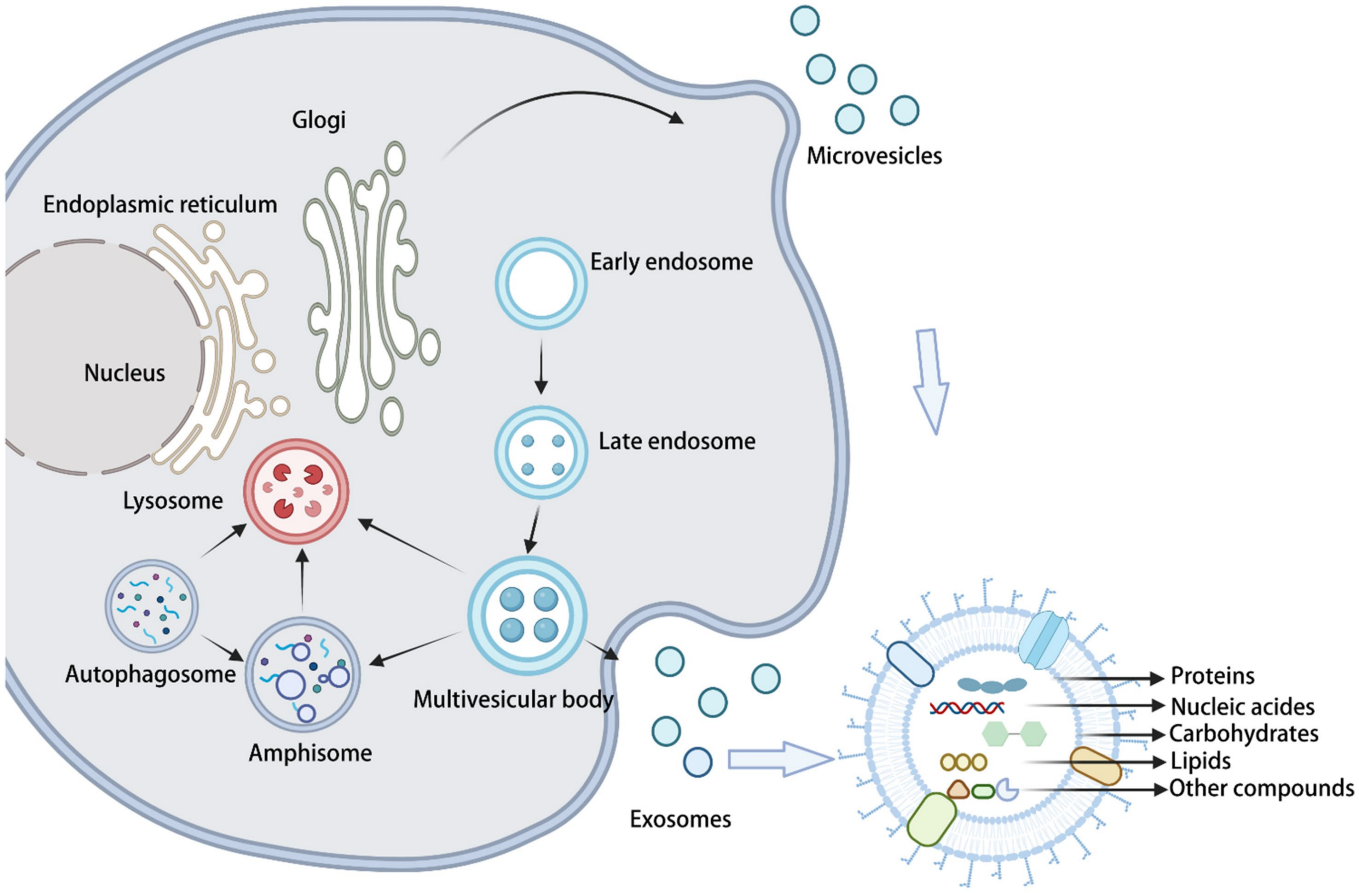
Biogenesis and composition of fungal sEVs.

Exosome formation (through the traditional Golgi pathway) begins with the formation of intracellular luminal vesicles (iLVs) by membranous organelles (e.g., endoplasmic reticulum, Golgi apparatus, and others), followed by the fusion of iLVs into multivesicular bodies (MVBs). Then, MVBs form vesicles of various sizes in an outgrowth manner. Finally, MVBs fuse with the cell membrane to release the vesicles into the extracellular space (Shao et al., 2018; Figure 1). On the other hand, microvesicles originate from the direct outward budding of the plasma membrane, generating a population of EVs with heterogeneous sizes. Apoptotic vesicles are also produced from the cell surface; however, they are only released by dying cells during their fragmentation. Recently, research advances have shown that specific fungal EVs are secreted via unconventional secretory pathways, including the Golgi reorganization and accumulation protein (GRASP) pathway, autophagy, the intracellular vesicle clusters (IVC) pathway, and endocytosis. GRASP maintains the Golgi structure and flows between different cisterns with Golgi-fused vesicles. Autophagosomes with IVC are not associated with the traditional secretory pathway. Endocytosis is accomplished through the sequential recruitment at endocytic sites of the proteins driving cargo sorting, membrane invagination, and vesicle release (De Toledo et al., 2019; Rizzo et al., 2020a). The mechanism by which fungal EVs cross the cell wall and are released into the extracellular environment is still unknown. Currently, there are three main working hypotheses to explain this process. According to the first hypothesis, the accumulation of EVs between the cell membrane and the cell wall generates a directional swelling pressure that forces EVs to cross the cell wall toward the extracellular space. In this case, the size of the EVs would be determined by the size of the wall pore and/or the thickness of the cell wall. According to the second hypothesis, cell wall remodeling provides access to the extracellular environment, through the action of degradative and synthetic enzymes associated with EVs. The third hypothesis postulates that protein channels (or structural cables) can direct EVs into the extracellular environment. Proteomics data suggest that some fungal EVs contain microtubulin or actin, which are components of structural cables (Brown et al., 2015).

## 3. Composition of fungal EVs

Initially, EVs were thought to be “garbage bags” for cells to remove debris or specific macromolecules (Jiang et al., 2022). Nevertheless, an increasing number of studies have demonstrated that fungal EVs contain a variety of bioactive substances, mainly proteins, nucleic acids, lipids, and carbohydrates. These molecules bind to the surface of the bilayer on the inner side of the vesicle, protecting these molecules from any external interference. With a better understanding of fungal EVs, the role and mechanisms of their carrier components in the physiological regulation of the organism and in disease development have been elucidated in detail. In this section, we outline general information on the composition of fungal EVs.

### 3.1. Proteins

The proteins in fungal EVs mainly include cytoplasmic and membrane-associated proteins. They also include various types of enzymes, which can increase intercellular communication, by secreting proteins to the surrounding environment. From recent studies, it became established that the plasma membrane proteins Sur7 and EVp1, the actin-binding protein Apb1, and the cell wall mannosylated protein are present in *Candida albicans* EVs (Nimrichter et al., 2016; Dawson et al., 2020). Proteomic analysis from different fungal EVs revealed the presence of virulence factors and a large number of proteins involved in biofilm formation, pathogenicity, and host-cell interactions (Sun et al., 2020; Zarnowski et al., 2021; Kulig et al., 2022). Proteins involved in vesicle biogenesis, or in the regulation of its size, production, and cargo, were also identified in a mouse model of aflatoxin endophthalmitis (Gandhi et al., 2022a). In recent years, the exploration of fungal virulence-associated proteins has become a hot topic of research. *Cryptococcus* EVs include heat shock protein (HSP70, etc.), hospholipase B, urease, and laccase (Marina et al., 2020; Park et al., 2020; Parreira et al., 2021; Jung et al., 2022). Analysis of *Histoplasma capsulatum* vesicles showed the presence of catalase B and superoxide dismutase precursors in these vesicles (Albuquerque et al., 2008). Proteins with antifungal effects, such as histone G and aspergillin, were found in EVs released by *Aspergillus fumigatus*-triggered polymorphonuclear granulocytes, which inhibited mycelial extension and affected fungal growth (Shopova et al., 2020). Recent studies have established that proteins from *Penicillium marneffei* and *Paracoccidioides brasiliensis*-derived vesicles are classified into six categories, based on their functions. These functions include protein/amino acid metabolism, stress/immune response, signal transduction/vesicle formation, carbohydrate/lipid metabolism, cellular organization/biogenesis, and others (Vallejo et al., 2012a; Yang et al., 2020).

### 3.2. Nucleic acids

Many studies have shown that fungal-derived EVs contain nucleic acids, including RNA (ribonucleic acid) and DNA (deoxyribonucleic acid), which have many different functions. A variety of RNA molecules are present in fungal EVs, including ncRNA, mRNA, snoRNA, snRNA, tRNA, microRNA, miRNA, and asRNA (De Toledo et al., 2019; Munhoz da Rocha et al., 2020). Less extensive research has been conducted on their DNA content, which remains to be explored in depth. EVs mediate nucleic acid export, participate in fungal intercellular communication, and in other cellular processes. *Cryptococcus neoformans*, *Paraccidioides brasiliensis*, *Candida albicans*, and EVs from *Saccharomyces cerevisiae* could export RNA molecules with different properties and biological functions, including interstrain virulence transfer in *Cryptococcus* (Peres da Silva et al., 2015; Marina et al., 2020). Cellular small RNA levels differed before and after treatment with the antifungal drug caspofungin. This observation provided useful material for the regulation of gene expression and intrinsic multidrug resistance mechanisms in *Candida auris*. Differences in virulence among distinct fungal isolates have been related to the RNA composition and content of the respective EVs. Moreover, RNAs from *Candida auris* EVs were shown to play a role in drug-induced stress signaling and in cell wall maintenance (Peres da Silva et al., 2019; Munhoz da Rocha et al., 2021; Zamith-Miranda et al., 2021; Amatuzzi et al., 2022). In addition, bioinformatic analysis of small RNAs carried by *Malassezia* EVs showed that these RNAs were not dependent on the biogenesis pathway of the RNAi (Rayner et al., 2017). Several studies have shown that EVs have a different RNA composition from their originating fungal cells. For example, one of the most enriched transcripts in EV exhibits low levels of expression in cells (CNAG_06651 amidohydrolase). there are 73 different ncRNA sequences in *Histoplasma capsulatum* and EVs of strain G186AR (Peres da Silva et al., 2018; Alves et al., 2019). These results reveal the specificity of nucleic acid transport in fungal vesicles.

### 3.3. Lipids

Lipids are important components of fungal EVs. The biogenesis of *Aspergillus fumigatus* EVs includes the formation of vesicles at the cell membrane level (Rizzo et al., 2020b). There is evidence that the lipid composition of fungal EVs also differs from that of its originating cell. For example, comparing the lipid composition of EVs from *Candida albicans* and *Candida auris* to the respective whole-cell composition indicates an enrichment of conserved hexosylceramide (HexCer, a lipid substance) in EVs of 67- and 109-fold, respectively (Zamith-Miranda et al., 2021). Therefore, the lipid composition could be a key factor to elucidating the EV biogenesis mechanism. Lipid components of fungal vesicles have the potential to modulate the interaction of fungi with their hosts. It has been shown that fatty acid and triglyceride-rich vesicles extracted from *Paraccidioides brasiliensis* induced a strong granulomatous response in mice (Alves et al., 1987). In *Cryptococcus neoformans* EVs, phosphatidylcholine (PC), phosphatidylethanolamine (PE), and sphingomyelin (SM) were the most differentially expressed lipids among the lipidomics associated with *Cryptococcus* infection. The deletion of the sterol biosynthetic gene *Erg6* induced changes in the lipid and protein content of *Cryptococcus neoformans* EVs, suggesting a role for sterols in their formation (Oliveira et al., 2020; Zhang et al., 2021). In addition, the lipid profiles of EVs cultured in different media were clearly different. Moreover, differences in lipid composition caused by growth conditions may affect virulence and immune potential (Cleare et al., 2020).

### 3.4. Other compounds

Fungal EVs contain a variety of biologically active substances, including cytochromes, carbohydrates, and other compounds. *Cryptococcus neoformans* produces vesicles containing podoconjugates (the main virulence factor of *Cryptococcus neoformans*) which are composed of two polysaccharides, glucuronosyl xyloglucan (GXM) and galactomannan (GalXM; LaRocque-de-Freitas et al., 2018; Marina et al., 2020). The carbohydrate metabolites found in *Histoplasma capsulatum* EVs are also potentially interesting. These compounds have a different glucose content and function in *Histoplasma capsulatum* EVs cultured in different media sources, possibly due to increased synthesis of glucose in fungal cells or differences in the EV biogenesis mechanism (Cleare et al., 2020). EVs also act as vehicles for the transmission of pathogens, including prions, protein infection factors, and others. Prions from *Saccharomyces cerevisiae* sup35 could be vertically and horizontally transferred between yeast cells, causing transmissible spongiform encephalopathy (TSE; Kabani and Melki, 2016; Liu et al., 2017).

## 4. Biological functions of EVs

**Figure 2:**
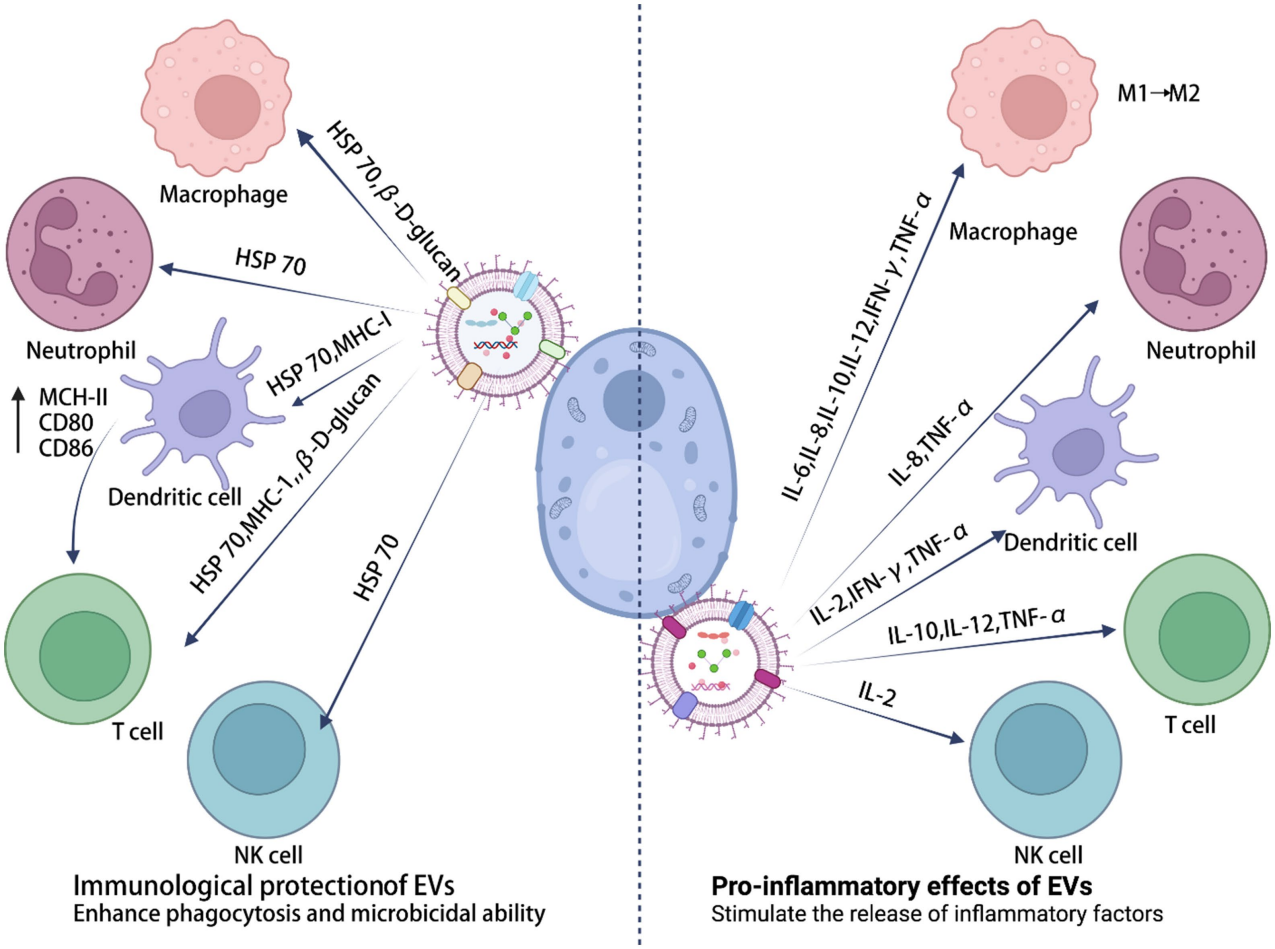
The immune potential of sEVs in fungal infections.

EVs are secreted by almost all cells and can be extracted from a wide range of tissue and cell culture supernatants. They can also be isolated from various body fluids, including urine, saliva, synovial fluid, bile, plasma, amniotic fluid, breast milk, semen, and ascites (Yáñez-Mó et al., 2015). EVs from several pathogenic fungi play a “double-edged sword” role in the regulation of the immune system (Figure 2). On the one hand, EVs have an important role in fungal infection and transmission, acting as mediators of cellular communication, to influence the interaction between fungal and host cells (e.g., macrophages, dendritic cells, and others), and to spread virulence factors into the host (Table 1). On the other hand, EVs have specific characteristics that induce protection, such as increased survival rates in *Cryptococcus*-infected *Galleria mellonella* (Reis et al., 2021), indicating their immunological potential (Table 2).

**Table 1:** The immune potential of EVs in fungal infections.

Types of fungal EVs	Mechanisms	Functions	References
*Candida albicans* EVs	*In vitro* effects, biofilm formation, and morphogenesis and attenuates virulence; *in vivo* reduces the fungal load of infected invertebrates	Immunological protection	Honorato et al. (2022)
*Candida albicans* EVs	Strains knocking out the CHO1 gene that affects lipid synthesis produce EVs that lack the ability to activate macrophages	Immunological protection	Wolf et al. (2015)
*Candida albicans* EVs	Proteins from mycelial EVs used to discover new diagnostic markers	Immunological protection	Martínez-López et al. (2022)
*Candida albicans* EVs	Aquaporin-5, CD5 antigen-like protein, gastrin, and vesicle-associated protein may serve as candidate diagnostic and prognostic markers	Immunological protection	Gandhi et al. (2022a,b)
*Candida albicans* EVs	Targeting this sialic acid transferase or inhibitory Siglecs during EVs-cell interactions could be a new therapeutic strategy to enhance antifungal response in patients	Immunological protection	Kobiela et al. (2022)
*Candida albicans* EVs	Sur7 and EVp1 can be candidate tetra-transmembrane protein markers	Immunological protection	Dawson et al. (2020)
*Candida albicans* EVs	The new antifungal drug turbinmicin disrupts the transport of EVs during biofilm growth and thus exhibits antifungal effects	Immunological protection	Zhao et al. (2021)
*Candida auris* EVs	Induces activation of phagocytes and increases phagocytosis, thereby regulating host cell defense mechanisms	Immunological protection	Zamith-Miranda et al. (2021) and Chan et al. (2022)
*Saccharomyces cerevisiae* EVs	Activates receptors on immune cells and can be used as a novel vaccine material for immune cell maturation	Immunological protection	Higuchi et al. (2023)
*Cryptococcus neoformans* EVs	Δsgl1 *de novo* Cryptococcus mutant EVs delay the acute lethality of Galleria mellonella to Cryptococcus infection and can be used as a vaccination strategy	Immunological protection	Colombo et al. (2019)
*Cryptococcus neoformans* EVs	Vesicles contain proteins that can act as immunomodulators, indicating their potential as vaccines	Immunological protection	Rizzo et al. (2021)
*Cryptococcus gattii* EVs	Vesicular peptide (Ile-Pro-Ile) improves survival of Galleria mellonella infected with Cryptococcus	Immunological protection	Reis et al. (2021)
*Paracoccidioides brasiliensis* EVs	Gal-3 affects the destruction and internalization of EVs by macrophages and promotes host defense	Immunological protection	Baltazar et al. (2021)
*Sporothrix* EVs	The macrophages showed increased fungicidal activity (phagocytic index) after co-culture of EVs with macrophages	Immunological protection	Campos et al. (2021)
*Aspergillus fumigatus* EVs	Trigger PMN to release afev	Immunological protection	Rafiq et al. (2022)
*Aspergillus fumigatus* EVs	Reduces neutrophil infiltration into the lung and increases fungal clearance in the respiratory tract, and is synergistic with amphotericin B in the treatment of fungal diseases	Immunological protection	Souza et al. (2022)
*Aspergillus flavus* EVs	Enhance phagocytosis and killing power of macrophages	Immunological protection	Gandhi et al. (2022a)
*Aspergillus flavus* EVs	Protein profiles in EVs can generate marker molecules that describe information about physiological and disease conditions induced by fungal infection and serve as novel prognostic targets for fungal endophthalmitis	Immunological protection	Brauer et al. (2020)

**Table 2:** Pro-inflammatory effects of fungal EVs.

Types of fungal EVs	Mechanisms	Functions	References
*Candida albicans* EVs	Mediates host inflammatory response, mediates NO, IL-12, TGF-β and IL-10 production by macrophages; stimulates IL-12, IL-10 and TNF-α production by dendritic cells	Pro-inflammatory effects	Honorato et al. (2022)
*Candida albicans* EVs	Significantly elevated levels of IL-6, IL-1β, TNF-α and IFN-γ in extracellular vesicles released from mice with *Candida albicans* endophthalmitis	Pro-inflammatory effects	Gandhi et al. (2022b)
*Candida albicans* EVs	Activates the L-arginine/nitric oxide pathway, increases NO levels, and reduces intracellular reactive oxygen species (ROS) and apoptosis, promoting the growth of *Candida albicans* itself. Synergistically, the damaging effect on host cells was enhanced.	Pro-inflammatory effects	Wei et al. (2023)
*Candida albicans* EVs	The EVs released during biofilm growth can produce an adherent extracellular matrix that provides the fungus with a protective layer for antifungal drugs	Pro-inflammatory effects	Zarnowski et al. (2021)
*Candida glabrata*, *Candida tropicalis*, and *Candida parapsilosis* EVs	Induces pro-inflammatory responses in human immune cells and stimulates the secretion of TNF-α and IL-8	Pro-inflammatory effects	Karkowska-Kuleta et al. (2020) and Kulig et al. (2022)
*Candida auris* EVs	Enhanced proliferation of yeast cells within macrophages	Pro-inflammatory effects	Zamith-Miranda et al. (2021)
*Saccharomyces cerevisiae* EVs	Transport of pathogenic prions	Pro-inflammatory effects	Higuchi et al. (2023)
*Cryptococcus neoformans* EVs	Transmission of virulence factors, including heat shock protein, phospholipase B, urease, laccase, and polysaccharides	Pro-inflammatory effects	Marina et al. (2020), Park et al. (2020), Parreira et al. (2021), and Jung et al. (2022),
*Cryptococcus neoformans* EVs	Induces immune responses in macrophages that escape intracellular restriction	Pro-inflammatory effects	Zhang et al. (2021)
*Cryptococcus gattii* EVs	Multiplication in macrophages	Pro-inflammatory effects	Goulart et al. (2010)
*Cryptococcus gattii* EVs	Transport of toxic molecules, such as melanin, laccase, podoconjugates, etc.	Pro-inflammatory effects	De Sousa et al. (2022)
*Malassezia* EVs	Role in sensitization and maintenance of AE inflammation	Pro-inflammatory effects	Gehrmann et al. (2011)
*Malassezia* EVs	Induction of IL-4 and TNF-α responses in PBMC	Pro-inflammatory effects	Zhang et al. (2019) and Vallhov et al. (2020)
*Histoplasma capsulatum* EVs	Contains virulence-associated proteins such as C0NND7 and C0NBI7	Pro-inflammatory effects	Cleare et al. (2020)
*Paracoccidioides brasiliensis* EVs	Induces macrophage production of pro-inflammatory mediators	Pro-inflammatory effects	da Silva et al. (2016)
*Paracoccidioides brasiliensis* EVs	Attenuated EVs induce *in vitro* expression of fungal virulence characteristics and enhance infection in mice.	Pro-inflammatory effects	Octaviano et al. (2022)
*Penicillium marneffei* EVs	Stimulate macrophage production of ROS, NO, IL-1β, IL-6, IL-10 TNF-α, etc.	Pro-inflammatory effects	Yang et al. (2020)
*Sporothrix brasiliensis* EVs	Stimulate macrophage production of IL-12 and IL-6	Pro-inflammatory effects	Campos et al. (2021)
*Sporothrix brasiliensis* EVs	Induce an increase in the phagocytic index and fungal load in DC	Pro-inflammatory effects	Ikeda et al. (2018)
*Aspergillus fumigatus* EVs	Mediate β-glucan-stimulated IL-1α secretion by neutrophils	Pro-inflammatory effects	Ratitong et al. (2021)
*Aspergillus flavus* EVs	Induce macrophages to produce inflammatory mediators, such as NO, TNF-α, IL-6, and IL-1β	Pro-inflammatory effects	Brauer et al. (2020)

### 4.1. Immunological potential of EVs in the course of fungal infections

Fungal EVs affect the morphogenesis and biofilm formation capacity of *Candida albicans*, stimulating adaptive immune responses. It has been shown that *Candida albicans* EVs could *in vitro* modulate yeast to mycelial differentiation, thereby inhibiting biofilm formation and attenuating virulence. *Candida albicans* EVs treated for 24 h lost their ability to penetrate agar, reduced fungal load in invertebrate models of *Candida albicans* infection, and were nontoxic when inoculated into *Galleria mellonella* (Honorato et al., 2022). In addition, both the presence and absence of *Candida albicans* genes could have qualitative and quantitative effects on EV size, composition, and immunostimulatory phenotype. Genes involved in phospholipid synthesis could affect not only multiple secretory phenotypes, but also the release of EVs carrying microbial cargo. Factors affecting lipid synthesis are important aspects for future studies of secreted virulence factors (Wolf et al., 2015). The characteristics of *Candida albicans* filamentous cell-secreted EVs (HEV) were compared with yeast EVs (YEV). Interesting differences were observed in the proteomic analyses of the biogenesis and function of mycelial EVs. These were shown to differ from yeast EVs and could be used to discover novel diagnostic markers and targets for the treatment of *Candida albicans* infections (Martínez-López et al., 2022). To understand the pathogenesis properties of *Candida* in endophthalmitis, and to validate potential diagnostic and prognostic markers, EVs associated with transport, cell adhesion, membrane fusion, and cytoskeletal organization of differentially expressed proteins (such as aquaporin-5, CD5 antigen-like protein, Gasdermin and vesicle-associated proteins) were studied in a mouse model of *Candida albicans* and *Aspergillus flavus* endophthalmitis (Gandhi et al., 2022a,b). Under an atopic dermatitis (AD) setting, *Candida albicans* promoted altered glycosylation patterns in keratinocyte-derived EVs, to interact with inhibitory Siglecs on antigen-presenting cells. Strategies that target this pathway to enhance antifungal responses and limit pathogen transmission may provide new therapeutic options for AD cutaneous candidiasis (Kobiela et al., 2022). In *Candida albicans* EVs, the plasma membrane proteins Sur7 and EVp1 could be fungal equivalents of four transmembrane protein tags for EV studies (Dawson et al., 2020). The new antifungal drug turbinmicin disrupts the transport of EVs during biofilm growth and thus exhibits antifungal effects (Zhao et al., 2021). *Candida auris* EVs induced the activation of phagocytes and increased phagocytosis, thereby regulating host cell defense mechanisms (Zamith-Miranda et al., 2021; Chan et al., 2022). *Saccharomyces cerevisiae* EVs activated receptors on immune cells and could be used as a novel vaccine material for immune cell maturation (Higuchi et al., 2023).

*Cryptococcus neoformans* mutants lacking the gene coding for steryl glucosidase (*Δsgl1*), enriched in sterol glucosidase-catalyzed steryl glucoside (SGs), and containing polysaccharide glucuronide oxyglycans (GXM) extracellular vesicles could delay the acute lethality of *Galleria mellonella* infection to *Cryptococcus neoformans*. This observation demonstrated the potential use of Δsgl1 EVs as a vaccination strategy for *Cryptococcus neoformans* (Colombo et al., 2019; Rizzo et al., 2021). A vesicular peptide (isoleucine-proline-isoleucine, Ile-Pro-Ile) from EVs produced by *Cryptococcus gattii* improved the survival of *Galleria mellonella* infected by *Cryptococcus* (Reis et al., 2021). Immunization with EVs had a positive impact on mice infected with *Paracoccidioides brasiliensis*. Specifically, it induced the mobilization of activated T lymphocytes and natural killer T cells to the infected lungs, improved the production of pro-inflammatory cytokines and histopathological features, and reduced the fungal load (Baltazar et al., 2021). *Sporothrix brasiliensis* EVs co-cultured with macrophages could enhance the fungicidal activity (assessed through the phagocytic index) of the macrophages (Campos et al., 2021). *Aspergillus* EVs enhanced phagocytosis and macrophage cytotoxicity, triggering the release of a unique set of antifungal EVs (afEVs) by polymorphonuclear granulocytes (PMNs). Infection with differentiated PLB-985 neutrophil-derived extracellular vesicles limited the growth of *Aspergillus fumigatus* hyphae, which also demonstrated antifungal effects (Rafiq et al., 2022). As an immunological tool, pre-exposing mice to EVs from *Aspergillus fumigatus* induced the production of a fungal antigen-specific IgG-rich serum, reduced alveolar infiltration by neutrophils, prevented extensive lung tissue damage, and improved phagocytosis and fungal clearance (Souza et al., 2022). EVs released by *Aspergillus flavus* enhanced the phagocytosis and cytotoxicity of macrophages. In addition, proteins from these vesicles could produce marker molecules that provided information about the physiological and disease status induced by the fungal infection. These marker molecules could serve as novel prognostic targets for fungal fundus disease (Brauer et al., 2020; Gandhi et al., 2022a).

### 4.2. Pro-inflammatory effects of fungal extracellular vesicles

*Candida albicans* EVs can mediate the host inflammatory response. Short exposure of macrophages to EVs resulted in the internalization of these vesicles and in the production of nitric oxide (NO), IL-12, TGF-β, and IL-10. Similarly, dendritic cells treated with EVs produced IL-12, IL-10, and TNF-α (Honorato et al., 2022). Significantly elevated levels of IL-6, IL-1β, TNF-α, and IFN-γ were observed in EVs released from mice with *Candida albicans* endophthalmitis (Gandhi et al., 2022b). In addition, *Candida albicans* EVs activated the L-arginine/NO pathway to reduce the intracellular levels of reactive oxygen species (ROS) and apoptosis. This outcome was achieved by increasing NO levels and upregulating the expression of the NO dioxygenase gene *YHB1*, thereby promoting the growth of *Candida albicans*. During host cell infection, *Candida albicans* EVs synergistically enhanced the pathogenesis and destructive effects of the fungus on host cells, as well as in the RAW264.7, HOK, TR146, and HGEC cell lines (Wei et al., 2023). EVs originating from *Candida glabrata*, *Candida parapsilosis*, and *Candida tropicalis* induced pro-inflammatory responses in human immune cells and stimulated the secretion of TNF-α and IL-8 (Karkowska-Kuleta et al., 2020; Kulig et al., 2022). Moreover, *Candida auris* EVs enhanced yeast cell proliferation in macrophages, and *Saccharomyces cerevisiae* EVs were shown to transport pathogenic prions (Zamith-Miranda et al., 2021; Higuchi et al., 2023).

The EVs secreted by *Cryptococcus neoformans* could disseminate virulence factors, including heat shock protein (Parreira et al., 2021), phospholipase B (Jung et al., 2022), urease (Marina et al., 2020), laccase (Park et al., 2020), and polysaccharides (Jung et al., 2022). These EVs could also induce an immune response on macrophages, to escape intracellular restriction (Zhang et al., 2021). *Cryptococcus gattii* promoted intracellular multiplication in macrophages, by secreting EVs and disseminating virulence factors (such as melanin, laccase, and podoconjugates) into the host (Goulart et al., 2010; De Sousa et al., 2022). *Malassezia* released EVs that induced IL-4 and TNF-α reactions in peripheral blood mononuclear cells (PBMCs; Gehrmann et al., 2011). These could also promote IL-6 production, by participating in NF-κB-dependent signaling pathways (Zhang et al., 2019; Vallhov et al., 2020). *Histoplasma capsulatum* EVs contain several virulence-related proteins, such as peroxidase (C0NND7) and alkaline phosphatase (C0NBI7; Cleare et al., 2020). Attenuated *Paracoccidioides brasiliensis* EVs induced *in vitro* expression of fungal virulence traits and enhanced infection in mice. In addition, EVs from virulent *Paracoccidioides brasiliensis* stimulated RAW 264.7 cells and bone marrow-derived macrophages to express high levels of inflammatory mediators, specifically TNF-α, IL-6, MCP-1 and NO (da Silva et al., 2016; Octaviano et al., 2022). Incubation of *Penicillium marneffei* EVs with macrophages resulted in elevated expression levels of ROS, NO, and several inflammatory factors, including IL-1β, IL-6, IL-10, and TNF-α. Proteomic studies have shown that they contain a number of virulence factors, including HSP, MP1p, and peroxidase (Yang et al., 2020). EVs isolated from *Sporothrix brasiliensis* stimulated the production of IL-12 and IL-6 by macrophages and increased the phagocytic index and fungal load in DCs (dendritic cells). They also increased the levels of immunogenic components and proteins in EVs, which, according to proteomics data, could be related to their virulence (Ikeda et al., 2018; Campos et al., 2021). *Aspergillus fumigatus* EVs mediated the β-glucan-stimulated IL-1α secretion by neutrophils (Ratitong et al., 2021). *Aspergillus flavus* EVs induced the production of inflammatory mediators, such as NO, TNF-α, IL-6, and IL-1β, by macrophages (Brauer et al., 2020).

## 5. Interaction between fungal EVs and host cells and their immune regulation

### 5.1. Yeast and yeast-like bacteria

#### 5.1.1. Candida albicans

*Candida albicans* is a commensal microorganism that colonizes a high percentage of the skin and mucosal surfaces of healthy individuals. However, when the immune defenses of an individual are disrupted, this fungus can cause invasive diseases and bloodstream infections. These infections can threaten the health and lives of immunocompromised patients (Andriantsitohaina and Papon, 2020). Numerous studies have shown that *Candida albicans* EVs could mediate host immune and inflammatory responses, being also associated with drug resistance in this fungus. A brief exposure of macrophages to EVs resulted in the internalization of these vesicles and in the production of NO, IL-12, TGF-β, and IL-10. Similarly, dendritic cells treated with EVs produced IL-12, IL-10, and TNF-α. In addition, *Candida albicans* EVs induced the upregulation of CD86 and MHC-II (Vargas et al., 2015), indicating their potential role in modulating the innate immune response to the fungus.

In addition, *Candida albicans* EVs contain proteins, lipids, and RNAs with immunogenic and pathogenic properties. The *Candida albicans* EV plasma membrane proteins Sur7 and EVp1 can be fungal equivalents of four transmembrane protein tags that can be exploited for the study of mammalian EVs (Dawson et al., 2020). The gene encoding phosphatidylserine synthase (*CHO1*) in *Candida albicans* is involved in phospholipid biosynthesis. In comparison with wild-type strains, the cho1^Δ^/^Δ^ mutant strains produced EVs with different PC and protein contents, which could not activate the NF-κB signaling pathway in bone marrow-derived macrophages (Wolf et al., 2015). In addition, fungal cell walls allowed rapid changes in cell volume and the passage of large liposomal particles, such as EVs. Cell wall remodeling is a normal adaptive response of fungi to environmental changes and was shown to enhance fungal adaptation to these microenvironments (Childers et al., 2020).

*Candida albicans* EVs not only play an important role in modulating the host inflammatory response, but can also affect its drug resistance. EVs released during biofilm growth produced an adherent extracellular matrix, which provided the fungus with a protective layer against antifungal drugs (Taff et al., 2012; Zarnowski et al., 2021). *Candida albicans* EVs could regulate fungal virulence and biofilm formation (Honorato et al., 2022; Kulig et al., 2022). Reduced levels of EVs and matrix polysaccharides in ESCRT-deficient mutants greatly increased susceptibility to the antifungal drug fluconazole. Moreover, the addition of *Candida albicans* biofilm EVs reversed biofilm matrix accumulation and drug susceptibility in ESCRT mutants. As such, *Candida albicans* biofilm EVs could play a key role in substrate production and biofilm drug resistance (Zarnowski et al., 2018). These findings provide new ideas and directions for the development of novel antifungal drugs. A recently developed antifungal drug, turbinmicin, disrupted EV transport during biofilm growth and impaired the subsequent assembly of the biofilm matrix, thereby reducing fungal resistance and exhibiting a broad-spectrum antifungal activity (Zhao et al., 2021).

#### 5.1.2. Non-*Candida albicans*

Although *Candida albicans* is the most common species of the *Candida* genus causing human infections, the global epidemiology of candidiasis is currently changing. As such, we collectively refer to other *Candida* genera as *non-Candida Albicans* (NCA) genera (Giacobbe et al., 2020). EVs from *Candida glabrata*, *Candida tropicalis*, and *Candida parapsilosis* induced pro-inflammatory responses in the THP-1 human monocyte cell line, stimulating the secretion of TNF-α and IL-8, and being involved in fungal infection and transport of virulence factors (Karkowska-Kuleta et al., 2020; Kulig et al., 2022). The prevalence of *Candida glabrata*, the second most common cause of invasive candidiasis, is currently increasing; however, the mechanisms by which it survives to the host’s antimicrobial and immune response remain unclear. Recent studies have demonstrated that phosphatidylinositol 3-kinase (PI3K), which is mainly involved in vesicle-mediated translocation and autophagy, is an important factor in the regulation of host immune responses, intracellular survival, and virulence in *Candida glabrata* (Rai et al., 2012, 2015). *Candida auris*, known as a “super fungus,” is prone to nosocomial outbreaks and has shown multiple drug resistance. *Candida auris* EVs regulated host cellular defense mechanisms, affecting adhesion to epithelial cells, intracellular cytotoxicity by macrophages, and the activation of dendritic cells (Zamith-Miranda et al., 2021). Application of the first-line antifungal agent caspofungin could increase the production levels of EVs, and also alter the types and amounts of their RNA and protein contents, which induced resistance in *Candida auris* (Chan et al., 2022).

*Saccharomyces cerevisiae* is a unicellular eukaryotic organism with many advantages for experimentation, such as rapid growth rates, a clear background, and easy biochemistry and molecular biology manipulation. As such, it is widely used as a natural carrier of gene therapy drugs and immune substances. Notably, the first genetically engineered vaccine, a recombinant hepatitis B vaccine, was produced using *Saccharomyces cerevisiae* (Yang et al., 2021). Recently, it was found that EVs from *Saccharomyces cerevisiae* could be used as a novel vaccine material (Higuchi et al., 2023). EVs also transport pathogenic prions and are vertically and horizontally transferred between yeast cells. Moreover, the number, size, morphology, and proteomic profile of yeast EVs were altered in strains with selective knockouts of ESCRT components. These observations suggest an important role of EVs in intercellular communication (Liu et al., 2017; Zhao et al., 2019). The EV biogenesis requires both conventional Golgi-derived pathways and unconventional secretory pathways, demonstrating the complexity of fungal cell biology (Oliveira et al., 2010). The yeast Eps15-like proteins Pan1p and Ent1p/2p synergistically regulated the interaction between endocytic vesicles and actin cables (Yoshida et al., 2021).

#### 5.1.3. *Cryptococcus* spp.

*Cryptococcus* is a deep fungus which, despite being exclusively aerobic, is a serious threat to human life and health. It is capable of infecting both immunocompromised and immunocompetent individuals (Ellis et al., 2019; Baddley et al., 2021). The main cryptococci causing human infections are *Cryptococcus neoformans* and *Cryptococcus gattii*. Central nervous system cryptococcosis is the most common form of cryptococcal infection and has a serious prognosis with high morbidity and mortality rates (Alves Soares et al., 2019; Xu et al., 2020).

##### 5.1.3.1. Cryptococcus neoformans

*Cryptococcus neoformans* is a parthenogenic intracellular pathogen that is most notably characterized by a surface envelope of polysaccharide pods. These structures are composed of two main polysaccharides, glucuronoxylated mannans (GXM) and galactosylated mannans (GalXM; LaRocque-de-Freitas et al., 2018). Numerous studies have shown that EVs secreted by *Cryptococcus neoformans* could induce immune responses (Zhang et al., 2021) and disseminate virulence factors into the host, including superoxide dismutase, phospholipase B, urease, and laccase (Cox et al., 2000). During the phenotypic characterization of *Cryptococcus* mutants lacking expression of the eukaryotic gene *NOP16*, this gene was found to be required for EVs production. The lack of *NOP16* gene expression resulted in, not only reduced EV numbers, but also different small molecule compositions (Castelli et al., 2022). The galactose lectin Galectin-3 (Gal-3) protein regulates host innate and adaptive immunity. Increasing Gal-3 levels during infection could inhibit fungal growth by inducing vesicle rupture and disrupting the delivery of virulence factors (Almeida et al., 2017).

EVs released from *Cryptococcus neoformans*-activated macrophages mediated pro-inflammatory signals, both *in vivo* and *in vitro*. Moreover, they might also be involved in immune response regulation, by transferring antigens to receptor cells or by activating immune-related pathways (Zhang et al., 2021). The interaction of these EVs with alveolar macrophages is essential for the control of infection. The ingestion of nascent *Cryptococcus* leads to its intracellular replication and to the appearance of polysaccharide podocyte-containing vesicles in the macrophage cytoplasm. This phagocytosis process involves permeabilization of the lysosomal membrane and secretion of intracellular podocyte polysaccharides into the vesicles (Steenbergen et al., 2001; Tucker and Casadevall, 2002). *Cryptococcus neoformans* have a unique intracellular pathogenic mechanism. It involves cytoplasmic accumulation of polysaccharide-containing vesicles and their intracellular replication, leading to the formation of phagosomes. Alvarez et al. first reported the phenomenon of “phagosome extrusion,” in which nascent *Cryptococcus* was shown to leave macrophages under conditions of phagosome maturation, actin depolymerization, and homotrimeric phagosome fusion. As Lei demonstrated, cryptococcal pathogens could escape the intracellular confinement of mammalian macrophages to continue their spread and proliferation (Alvarez and Casadevall, 2006).

Liposomes containing amphotericin B could penetrate the fungal cell wall, which has deformable viscoelasticity to deliver amphotericin B to the cell membrane. This observation implies that the cell wall allows for a transmural vesicle flow. It was also shown that *Cryptococcus neoformans* EVs were able to cross the cell wall and deliver soluble membrane-bound effectors and other molecules to the extracellular space (Walker et al., 2018). The accumulation of sterol glucosides (SGs) has an effect on the glucuronide glucan (GXM) in fungal pod membranes. *Cryptococcus neoformans* mutants lacking the gene coding for sterol glucosidase (*Δsgl1*) were shown to be nonpathogenic. The deletion of Erg6, which is involved in fungal ergosterol biosynthesis, also affected *Cryptococcus neoformans* virulence and failed to induce protection in a vaccination model. GXM-containing EVs enriched in SGs delayed the acute lethality of *Galleria mellonella* against *Cryptococcus neoformans* infection (Colombo et al., 2019). A well-characterized vesicle transport adapter, the AP-3 complex, was shown to selectively transport the Arf1-GTPase and the Yck3 kinase to the vesicle membrane. These processes resulted in vesicle/lysosomal membrane permeabilization and the molecular triggering of cell death (Stolp et al., 2022).

##### 5.1.3.2. Cryptococcus gattii

*Cryptococcus gattii* can be a causative agent of disease in immunocompetent individuals. Likewise, it can multiply within macrophages, in a process associated with intracellular replication and cytoplasmic accumulation of polysaccharide-containing vesicles (Goulart et al., 2010). *Cryptococcus* EVs have multiple biological roles related to the transport of various important virulence molecules, such as melanin, laccase, podoconjugates, and others (De Sousa et al., 2022). Genes coding for proteins involved in vesicle formation and transport may be associated with virulence. Representative difference analysis (RDA) was used to identify *Cryptococcus neoformans* and *Cryptococcus gattii* genes differentially expressed during growth in rat intraperitoneal macrophages. Upregulated genes in *Cryptococcus gattii* were found to be associated with cell growth, aerobic respiration, and protein binding (Goulart et al., 2010).

There is a unique “division of labor” mechanism in the pathogen population, in which specific cells adopt a dormant behavior, in order to support the growth of neighboring cells. Bielska et al. demonstrated that a pathogenic “division of labor” process could be triggered at large cellular distances and mediated by the release of EVs by the fungus (Bielska et al., 2018). Fungal EV molecules are characterized by induced protection. EVs obtained from *Cryptococcus gattii* were analyzed, in which a vesicular peptide (isoleucine-proline-isoleucine, Ile-Pro-Ile) improved the survival of *Galleria mellonella* infected by *Cryptococcus gattii* or *Cryptococcus neoformans* (Reis et al., 2021). It is suggested that the proteins in EVs produced by *Cryptococcus gattii* have an immunological potential. Not only the *Cryptococcus* Zip3 gene, a homolog of *Saccharomyces cerevisiae* Atx2p, affects the secretion of *Cryptococcus* EVs and GXM, but also the deletion of Zip3 leads to impaired virulence of *Cryptococcus gattii* (Garcia et al., 2020).

#### 5.1.4. Malassezia

*Malassezia* is a lipophilic fungus that parasitizes the skin surface of humans and other mammals (White et al., 2014). It has been recently reported that the development of specific lipid-deficient skin diseases, such as atopic eczema (AE), is related to *Malassezia* spp. Moreover, EVs released by *Malassezia* can also play a role in the sensitization and maintenance of AE-related inflammation (Johansson et al., 2018). Meanwhile, *Malassezia* EVs can release EVs carrying antigens, small RNAs, and allergens, which induce IL-4 and TNF-α responses in PBMCs (Gehrmann et al., 2011). It can also be internalized into keratin-forming cells and promote IL-6 production, by participating in NF-κB-dependent signaling pathways (Zhang et al., 2019; Vallhov et al., 2020). Bioinformatic analysis of vesicular small RNAs indicates that these RNAs are likely not dependent on the RNAi biogenesis pathway (Rayner et al., 2017). According to a recent report, *Malassezia globosa* inhibited *Staphylococcus aureus* biofilm formation, through the ability to secrete the MgSAP1 aspartyl protease (Ianiri et al., 2018). Whether *Malassezia* EVs are involved in beneficial outcomes for the host is a subject for further research.

### 5.2. Temperature biphasic fungi

#### 5.2.1. Histoplasma capsulatum

*Histoplasma capsulatum* is a common temperature biphasic fungus, which can produce different morphological features under different temperature conditions. It can also cause widely distributed granulomatous diseases. *Histoplasma capsulatum* produces EVs carrying lipids, proteins, nucleic acids, and polysaccharides of remarkable biological significance (Albuquerque et al., 2008; Rodrigues et al., 2008; Reis et al., 2019). *Histoplasma capsulatum* EVs contain multiple virulence-related proteins, such as peroxidase (C0NND7) and alkaline phosphatase (C0NBI7; Cleare et al., 2020). In comparison with EVs from *Candida albicans*, *Cryptococcus neoformans*, and *Saccharomyces cerevisiae*, the RNA content and class of *Histoplasma capsulatum* EVs are different (Alves et al., 2019). Monoclonal antibodies (mAb) could differentially regulate the loading and release of EVs by *Histoplasma capsulatum* in both concentration-dependent and independent manners, leading to different effects on host immune effector mechanisms (Matos Baltazar et al., 2016; Baltazar et al., 2018).

#### 5.2.2. Paracoccidioides brasiliensis

*Paracoccidioides brasiliensis* could invade the mucous membranes, skin, and lungs, and cause chronic pyogenic granulomatosis. EVs from *Paracoccidioides brasiliensis* contain proteins, lipids, and RNAs. These molecules could induce the production of pro-inflammatory mediators by mouse macrophages (in a dose-dependent manner) and also modulate the innate immune response (da Silva et al., 2016). Protein characterization of EVs from the pathogenic stage of the *Paracoccidioides brasiliensis* yeast revealed multiple protein functions. These functions were mostly related to protein and carbohydrate metabolism, stress response, signaling pathways, cell division, transport, and additional potentially unclear functions (Vallejo et al., 2012a). Differences in virulence among fungal isolates could be related to their distinct EV-RNA content. These EVs might modulate the transcriptome of dendritic cells (Peres da Silva et al., 2019). Consistently, Vallejo observed differences in sterol composition and also between EVs and whole cells (Vallejo et al., 2012b).

Gal-3 affects the destruction and internalization of *Paracoccidioides brasiliensis* EVs by macrophages. It is also beneficial to mammalian hosts, by promoting host defense during infection (Hatanaka et al., 2019). In contrast, attenuated *Paracoccidioides brasiliensis* EVs induced *in vitro* expression of fungal virulence traits and enhanced infection in mice (Octaviano et al., 2022). The proteins from *Paracoccidioides brasiliensis* EVs could alter cell wall composition and affect fungal-host cell interactions. They could also release immunoreactive compounds into the extracellular environment, thereby affecting fungal pathogenesis. Therefore, the biosynthesis of EVs is a potential target for the development of novel antifungal drugs and vaccines (Nimrichter et al., 2016).

#### 5.2.3. Penicillium marneffei

*Penicillium marneffei* is an opportunistic fungus that invades and proliferates within macrophages. It frequently causes disseminated infections in patients with AIDS. Incubation of EVs with macrophages leads to increased expression levels of ROS, NO, and several inflammatory factors, including IL-1β, IL-6, IL-10, and TNF-α. A study on the protein composition of EVs from *Penicillium marneffei* identified a total of 394 species, which were shown to be mostly involved in protein/amino acid metabolism and signal transduction. The proteins from these EVs were mainly localized in the cytoplasm, ribosomes, and nucleus. Most of the proteins were closely related to other members in the protein–protein interaction network of TM-derived EVs (Yang et al., 2020).

#### 5.2.4. Sporothrix

*Sporothrix* can spread through the bloodstream and cause nodules and abscesses in the skin throughout the body. It has been established that ascomycetes, such as *Sporothrix schenckii*, *Candida albicans, Candida parapsilosis*, and *Saccharomyces cerevisiae* can produce EVs (Albuquerque et al., 2008). In addition, fungal EVs can facilitate fungal spread, by delivering essential proteins, lipids, nucleic acids, and some immunologically active components. EVs from *Sporothrix brasiliensis* have more immunologically active components, in comparison with those from *Sporothrix schenckii*, which could explain the greater virulence of *Sporothrix brasiliensis* (Ikeda and Ferreira, 2021). Consistently, macrophages at different concentrations showed increased fungicidal activity (as assessed by their phagocytic index) when co-cultured with *Sporothrix brasiliensis* EVs. Moreover, the levels of IL-12 and IL-6 levels were dose-dependently increased during infection. Molecules that are important in the immune response, such as MHC class II and immunoglobulin CD86, were overexpressed in *Sporothrix brasiliensis*-infected mice (Campos et al., 2021). These molecules showed that EVs could play a protective role during infection with *Sporothrix brasiliensis*. In addition, EVs isolated from *Sporothrix brasiliensis* induced an increase in the phagocytic index and fungal load in DCs, which, according to proteomics, could be related to the presence of virulence-associated immunogenic components and proteins in EVs (Ikeda et al., 2018).

### 5.3. Aspergillus

#### 5.3.1. Aspergillus fumigatus

*Aspergillus fumigatus* is a conditionally pathogenic fungus that is widely present in the natural environment. When inhaled by humans, it can cause *Aspergillus* allergy-related diseases. Rizzo et al. first characterized fungal EVs in a protoplast model consisting of *Aspergillus fumigatus* cells without the cell wall. Protoplast EVs, which contain bioactive components, such as lipids, glycans, and proteins, could be a promising model for functional studies of fungal EVs (Rizzo et al., 2020b). UV irradiation of *Aspergillus fumigatus* EVs resulted in reduced colony formation levels, as well as in the upregulation of the *MPKC* stress response gene. These observations identified a role for EVs in fungal intraspecific communication. Thus, understanding the mechanism of EVs-fungal interaction can be useful for antifungal therapy (Bitencourt et al., 2022).

*Aspergillus fumigatus* EVs mediated the β-glucan stimulation of IL-1α secretion by neutrophils (Ratitong et al., 2021). Recently, it has been reported that *Aspergillus fumigatus* triggered the release of a unique set of antifungal EVs (afEVs) by PMNs. A proteomic analysis has shown that afEVs were rich in bioactive antifungal proteins that stimulated the production of inflammatory mediators and could lead to fungal clearance (Shopova et al., 2020). Multiple immunizations with EVs from *Aspergillus fumigatus* reduced neutrophil infiltration into the lungs, increased fungal clearance in the airways, and reduced lung injury. These effects were also related to a synergistic effect in the treatment of pulmonary aspergillosis infection with amphotericin B. This observation suggests that EVs can induce the activation of the host immune system and generate a protective response against *Aspergillus fumigatus* infection (Souza et al., 2022).

#### 5.3.2. Aspergillus flavus

*Aspergillus flavus* is the second most common cause of aspergillosis worldwide. EVs from *Aspergillus flavus* could *in vitro* induce the production of inflammatory mediators, such as NO, TNF-α, IL-6, and IL-1β by macrophages, enhancing phagocytosis and cytotoxicity by these cells. Pre-stimulation with *Aspergillus flavus* EVs in wax borer larvae triggered an immune response and facilitated fungal clearance. This observation indicates that *Aspergillus flavus* EVs are biologically active and can stimulate the innate immune system (Brauer et al., 2020). EVs released from aflatoxin-infected retinal pigment epithelium (RPE) cells could also activate immune signaling pathways and might contribute to the pathogenesis of endophthalmitis, for which proteins in EVs may serve as clinical diagnostic and prognostic biomarkers (Gandhi and Joseph, 2022; Gandhi et al., 2022a).

## 6. Prospect and outlook

### 6.1. Drug carriers

**Figure 3:**
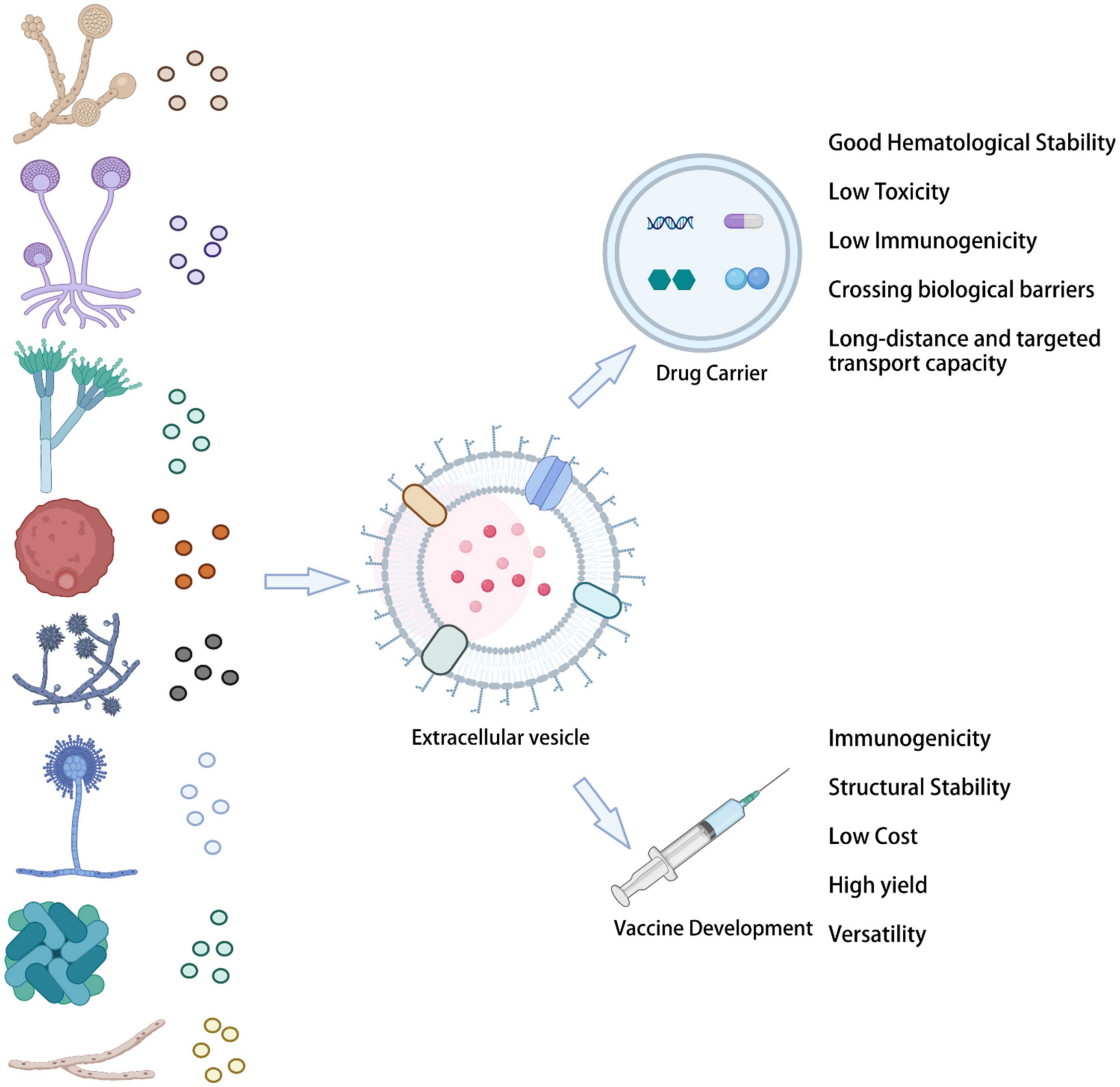
Fungal EVs in the clinical setting.

Fungal-derived vesicles provide numerous outstanding advantages for the delivery of biological drugs. Specifically, they show unparalleled superiority as drug carriers, enhancing the solubility of insoluble molecules and their bioavailability, reducing the dose, and providing efficient drug targeting. In addition, bioengineered EVs offer advantages as drug delivery carriers: they have excellent hematological stability and biocompatibility, low toxicity and immunogenicity, as well as a long-distance and targeted transport capability, being also able to cross biological barriers (Figure 3).

Encapsulation of griseofulvin in deformable membrane vesicles (DMVs) for dermal administration has high drug permeability and skin retention, which can overcome its low bioavailability for oral administration. However, this approach also has many side effects and requires a long duration of the treatment (Indian patent application 208/DEL/2009; Aggarwal and Goindi, 2012). Multimolecular liposome/anionosomes containing clotrimazole (the vesicle gel system) were prepared by lipid hydrolysis to provide sustained and controlled release of appropriate drugs for local vaginal therapy (Ning et al., 2005). In addition, an effective ocular nano-vesicle carrier, which provides prolonged and controlled ocular delivery of clotrimazole (CLT) to the carrier system, has also been developed (Basha et al., 2013). Moreover, a novel nanosystem vesicle loaded with itraconazole enhanced corneal permeation and antimycotic activity, to combat *Candida albicans* infections (Elmeshad and Mohsen, 2016).

### 6.2. Vaccines

Although EV studies are becoming widespread in different biomedical fields, in mycology, this area of research is still in its infancy. Fungal resistance faced in the antibiotic era has created an urgent need for developing more vaccine types against fungal diseases, which are estimated to affect more than 1 billion people worldwide, with significant mortality (Bongomin et al., 2017; Rizzo et al., 2020a). Fungal EV vaccines have the following advantages: first, their high production yield, due to the rapid replication rates of fungal cells and their ability to secrete EVs in large quantities. Second, their multifunctionality indicates the possibility of modifying multiple antigens. Third, the low complexity of their composition. Fourth, their high stability.

The presence of known protective antigens on the surface of EVs suggests their potential for vaccine development. For example, *Cryptococcus neoformans* EVs contain proteins from the Mp88, Cda, and Gox families, which have immunomodulatory effects. Moreover, EVs obtained from *Cryptococcus neoformans* mutant strains produced a strong antibody response in mice, greatly prolonging the survival time of animals infected with *Cryptococcus neoformans* (Rizzo et al., 2021). In the last few years, substantial progress has been made in the host-fungal relationship. Recurrent vulvovaginal candidiasis (RVVC) is characterized by recurrent annual episodes. The virosomal vaccine (PEV7) and the immunotherapeutic vaccine (NDV-3A) can be used to prevent RVVC (de Bernardis et al., 2012; Gil-Bona et al., 2015). Through a comparative proteomic study of *Candida albicans* and its soluble secretory proteins, EVs from *Candida albicans* were identified as a new vaccine source by Ana et al. Interestingly, the Bgl2 protein found in vesicles and supernatants without EVs had the potential to prevent systemic candidiasis in a mouse model (Edwards et al., 2018).

## 7. Conclusion

**Figure 4:**
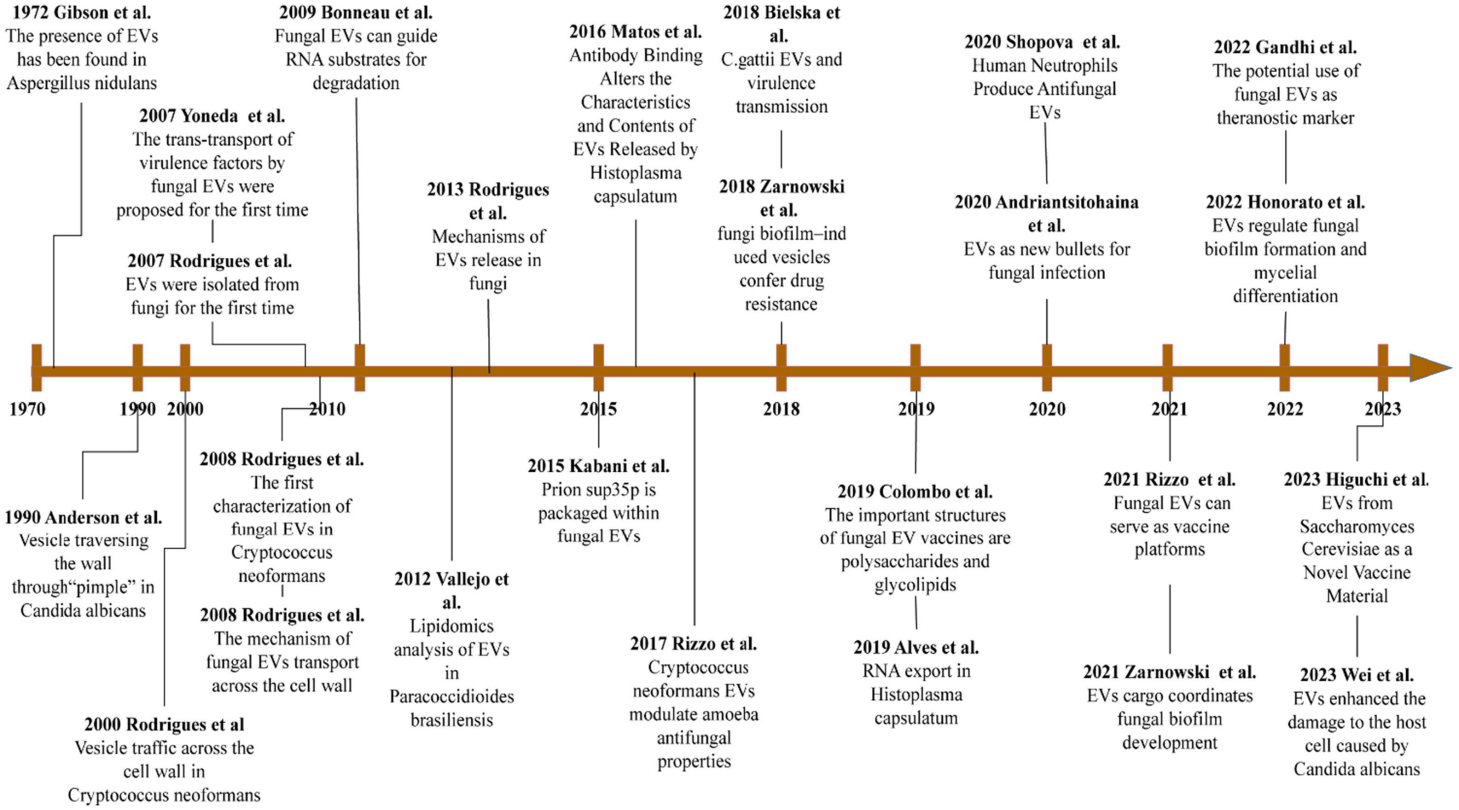
Important advances in the study of fungi EVs. This image references the image PMID: 32760680 and updates on it.

The study of fungal EVs has been neglected for many years, due to the thick and hard properties of the cell walls of fungi. However, according to recent findings, fungal EVs are important mediators of intercellular communication, allowing the functional transfer of bioactive molecules, which include lipids, proteins, and nucleic acids. Fungal EVs provide access to nutrients, toxin delivery, and cell adhesion. They also promote biofilm formation, to evade host defense systems. More importantly, fungal EVs characterize the induction of protection in fungal infections and have a remarkable potential for clinical diagnosis, treatment, and prevention. EVs represent a new generation of drug delivery systems, which offer high delivery capacity and efficiency, intrinsic targeting properties, and low immunogenicity. In recent years, a large amount of research has been directed toward developing safe and efficient bioengineered EVs (Figure 4).

However, there are still important limitations in the application of EVs as vaccines, such as: how to isolate fungal EVs with high efficiency and standardization, their scalable production, and storage of standard procedures. Due to our insufficient understanding of the mechanisms of EVs production, these are still urgent issues to be solved. In recent years, there has been an increase in vesicle production utilizing genetic engineering modifications (e.g., gene mutations, pathway modifications, and knockout of specific genes). Additional limitations to overcome include reducing the virulence of EVs and exploring their complex delivery mechanisms for precise delivery of antigens, as different pathogens express different virulence factors. In the near future, we need to develop vesicle materials with both improved safety and biodegradability. We also need to continuously optimize the vesicle preparation process. Such optimization will contribute to solving the limitations of their low encapsulation capacity, poor physical stability, low drug loading efficiency, and high cost of lipid-like vesicle production. We anticipate that, after continuous efforts, fungal EVs will provide a new direction for the development of its medical utilization.

## Author contributions

YL wrote the first draft alone. All authors contributed to the article and approved the submitted version.

## Conflict of interest

The authors declare that the research was conducted in the absence of any commercial or financial relationships that could be construed as a potential conflict of interest.

## Publisher’s note

All claims expressed in this article are solely those of the authors and do not necessarily represent those of their affiliated organizations, or those of the publisher, the editors and the reviewers. Any product that may be evaluated in this article, or claim that may be made by its manufacturer, is not guaranteed or endorsed by the publisher.
